# Mindfulness-based interventions for pain, anxiety, depression, and quality of life in patients receiving palliative care: a meta-analysis

**DOI:** 10.1177/17449871261419719

**Published:** 2026-02-26

**Authors:** Ice Septriani Saragih, Dame Elysabeth Tuty Arna Uly Tarihoran, Huey-Ming Tzeng, Ita Daryanti Saragih

**Affiliations:** Lecturer, Nursing Department, Diponegoro University, Semarang, Central Java, Indonesia; Associate Professor, School of Nursing, Universitas Kristen Krida Wacana Jakarta, Indonesia; Professor, School of Nursing, The University of Texas Medical Branch at Galveston, TX, USA; Postdoctoral Fellow, College of Information Sciences and Technology, The Pennsylvania State University, State College, PA, USA

**Keywords:** meta-analysis, mindfulness-based interventions, pain, palliative care, quality of life

## Abstract

**Background:**

Palliative care addresses not only physical symptoms but also emotional and spiritual suffering in patients with advanced disease. Mindfulness-based interventions (MBIs) may help alleviate symptoms and enhance quality of life. Previous reviews have explored MBIs’ efficacy, but evidence remains inconclusive.

**Objective:**

To systematically evaluate the effects of MBIs on pain, anxiety, depression, and quality of life in patients receiving palliative care.

**Methods:**

Six databases and grey literature were searched from databases inception to 22 May 2025. Outcomes included pain, anxiety, depression, and quality of life. Meta-analyses with forest plots estimated pooled standardised mean differences (SMDs). Egger’s test assessed publication bias, and leave-one-out sensitivity analysis evaluated result stability.

**Results:**

Thirteen trials were included. MBIs significantly reduced anxiety (SMD = −0.68, 95% CI = −1.15 to −0.21, *I*^2^ = 65.37%, *p* < 0.001) and depression (SMD = −0.43, 95% CI = −0.68 to −0.18, *I*^2^ = 0.00%, *p* < 0.001). Effects on pain and quality of life were less clear, with insufficient evidence to confirm significant benefit.

**Conclusions:**

MBIs appear to alleviate anxiety and depression in palliative care patients, highlighting their potential as supportive interventions. Further research is warranted to clarify effects on pain and quality of life.

## Introduction

Palliative care, as a form of active holistic care, is an approach aimed at improving the quality of life and alleviating suffering in patients with incurable and advanced diseases (Kostina et al., 2024; Nottelmann et al., 2021). This model of care addresses the physical, psychosocial, and spiritual needs of patients with life-limiting diagnoses (Teoli et al., 2019). Approximately 61 million people worldwide with life limiting diagnoses experience palliative care to alleviate physical and psychosocial distress (Kluger et al., 2023; Rosa et al., 2023). Access to palliative care has increased in recent years; however, significant challenges remain in integrating palliative care interventions with existing healthcare delivery (Amroud et al., 2021; Langley et al., 2024).

The gradual deterioration and emotional distress experienced by patients with incurable and advanced diseases have been addressed through pharmacological techniques to manage physical symptoms, such as pain (Coelho et al., 2017). However, palliative care extends beyond the relief of physical symptoms, namely, patients with advanced disease with emotional and spiritual distress may benefit from alternative non-pharmacological interventions aimed at alleviating symptoms and improving quality of life (Paley et al., 2023; van Veen et al., 2024). In recent years, the implementation of mindfulness-based interventions (MBIs) as a non-pharmacological approach in palliative care has become increasingly popular (Poletti et al., 2019a). MBIs are considered a promising strategy to enhance coping skills in patients with incurable and advanced diseases, aiming to improve symptom management and overall quality of life in palliative care settings (Stadnyk et al., 2023). MBIs, designed to alleviate symptoms of emotional and spiritual distress as well as physical pain, aim to foster greater attention and awareness of thoughts, feelings and perceptions while embracing the challenges of the present moment (Baer and Huss, 2008; Schuman-Olivier et al., 2020). Importantly, this emphasis on mindful presence and holistic awareness resonates strongly with foundational nursing theories. For example, Jean Watson’s Human Caring theory (Watson, 2009) holds that nursing is fundamentally a transpersonal, caring partnership in which authentic presence, emotional and spiritual support, and promotion of human dignity are central to healing (Watson, 1997, 2009). Watson’s framework enshrines compassion, active listening, being there and understanding as core caring-healing factors that foster healing for patients. In a similar vein, Cara and O’Reilly (2008) described how grounding nursing practice in humanistic caring values helps nurses and patients alike transcend meaninglessness and experience more gratifying, holistic care. Turkel et al. (2018) further argued that ‘caring science’ bridges empirical knowledge with the lived (epistemological and ontological) experience of care, ‘advancing the epistemology and ontology of carin’, (Turkel et al., 2018). In other words, MBIs, which are evidence-based practices delivered with mindful presence and compassion, embody exactly the kind of integrated, humanistic-scientific caregiving these theories advocate. Viewed through this lens, MBIs can be understood as authentic caring interventions in palliative nursing: they operationalise Watson’s transpersonal caring processes and related humanistic constructs by supporting patients’ inner strengths and dignity. Several types of MBIs have been applied to patients in palliative care settings, including mindfulness-based stress reduction (MBSR; Poletti et al., 2019b), mindful breathing (Guan et al., 2021) and mindful meditation (Miroslav, 2020).

Although previous studies have explored the efficacy of MBIs for patients receiving palliative care (Guan et al., 2021; Lim et al., 2021; Ng et al., 2016; Tan et al., 2023; Warth et al., 2020; Yik et al., 2020), only two systematic reviews have been conducted (Latorraca et al., 2017; Stadnyk et al., 2023). Although these reviews thoroughly analysed the efficacy of MBIs, the conclusions were deemed not robust enough to definitively establish the true effects of these interventions. For example, Stadnyk et al. (2023) systematically analysed individual studies but presented results narratively due to insufficient sample sizes and qualitative data, which were insufficient to conclusively determine the interventions’ true effects. Similarly, Latorraca et al. (2017) included only four individual studies in their investigation of MBIs on quality of life and perceived distress. Although a meta-analysis was performed, only one study per outcome could be synthesised due to the limited number of eligible trials, which substantially restricted the robustness and interpretability of their findings. In addition, their review did not capture the numerous RCTs published in subsequent years, including trials evaluating brief, digitally delivered or palliative-specific mindfulness interventions, resulting in an evidence base that is now considerably outdated. To address these limitations and strengthen the current understanding of MBIs in palliative care, a systematic review and meta-analysis incorporating a larger and more contemporary set of trials is needed. Therefore, the present study aimed to systematically synthesise evidence on MBIs specifically for pain, anxiety, depression and quality of life in patients receiving palliative care.

## Materials and methods

### Protocol and registration

The Preferred Reporting Items for Systematic Reviews and Meta-Analysis (PRISMA) statement was employed for this study (Page et al., 2021). The study protocol was registered in PROSPERO (Registration No. CRD42024570587).

### Literature search for identification studies

A systematic search for relevant trials was conducted from database inception to 22 May 2025, using six databases: CINAHL Plus with Full text, Cochrane Library, Embase, Medline, PubMed, and Web of Science. The Medical Subject Headings (MeSH) of PubMed, CINAHL Plus with Full text and term used in the previous reviews were used to develop the key terms with the help of a science librarian to validate the terms used (Latorraca et al., 2017; Stadnyk et al., 2023). Search strategies combined controlled vocabulary (e.g. MeSH and Emtree terms) and free-text keywords related to palliative or end-of-life care (e.g. palliative care, terminal care, end of life, dying patient), MBIs (e.g. mindfulness, meditation, MBSR, mindfulness-based cognitive therapy, yoga) and randomised study designs (e.g. randomised controlled trial (RCT), crossover trial, randomisation). Database-specific syntax and field tags (title/abstract/keywords) were applied as appropriate. Grey literature was additionally searched using Google Scholar, and reference lists of relevant reviews were screened to minimise the risk of missing eligible trials. No restrictions on language or geographic location were applied. Detailed search strategies are available in the Supplemental Appendix B.

### Eligibility criteria

The Population, Intervention/Issue of interest, Comparison, Outcome and Study design (PICOS) method was used to define the inclusion criteria of potential trials in this study (Amir-Behghadami and Janati, 2020). The inclusion criteria were the following: Population: patients receiving palliative care (no restriction on age, sex, disease, onset of disease, stage of disease); Intervention: MBI including mindfulness meditation, mindful movement, mindfulness in daily life, MBSR, mindfulness-based cognitive therapy and acceptance and commitment therapy; Comparison: treatment as usual, conventional therapy or usual care; Outcomes of interest were pain, anxiety, depression and quality of life, measured using validated quantitative scales that were amenable to meta-analysis; and Study design: RCT. RCTs in the form of a protocol or that did not provide the mean and standard deviation before and after the intervention delivered were excluded.

### Study selection and data extraction

**Figure 1.**
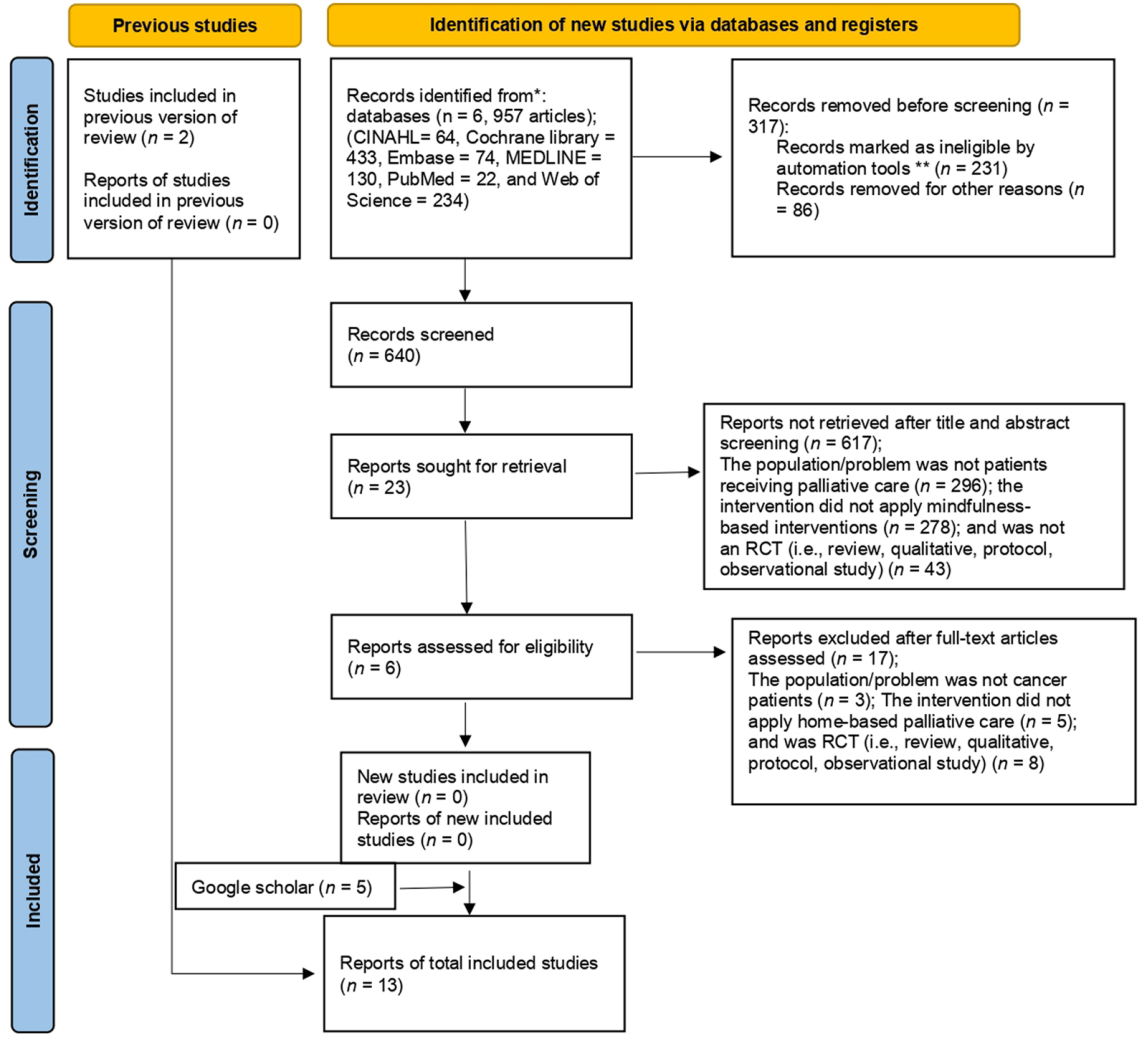
PRISMA flowchart diagram. *Source*. From Page et al. (2021). For more information, visit: http://www.prisma-statement.org/ *Consider, if feasible to do so, reporting the number of records identified from each database or register searched (rather than the total number across all databases/registers). **If automation tools were used, indicate how many records were excluded by a human and how many were excluded by automation tools.

Two authors independently performed the study selection using EndNote software version 21, Clarivate Plc, United Kingdom. After the first author removed duplicates using the ‘find duplicate’ tool, both authors screened all titles and abstracts based on the inclusion criteria. Upon obtaining relevant papers, the two authors held a meeting to resolve any discrepancies that arose during this process. The database search yielded 957 records across six databases. After removal of duplicates, 640 unique records remained and were screened at the title and abstract level. During this stage, 617 records were excluded: 296 did not involve patients receiving palliative care, 278 did not include MBIs, and 43 were not RCTs (e.g. reviews, qualitative studies, protocols or observational studies). Following this screening process, 23 studies were deemed potentially eligible and retrieved for full-text review. Of these, 17 studies were excluded because they did not involve cancer patients (*n* = 3), did not apply home-based palliative care (*n* = 5) or were not RCTs (*n* = 8). After full-text assessment against the inclusion criteria, six studies met all eligibility requirements and were included in the analysis. To further ensure the comprehensiveness of the search, additional grey literature searches were conducted using Google Scholar, identifying five additional eligible studies. Reference lists of relevant papers were also screened, yielding two further studies. In total, 13 studies were included in the final analysis (Bower et al., 2021; Galfin et al., 2012; Guan et al., 2021; Kubo et al., 2024; Lim et al., 2021; Look et al., 2021; Ng et al., 2016; Speca et al., 2000; Tan et al., 2023; Teo et al., 2020; Warth et al., 2015; Warth et al., 2020; Yik et al., 2020). The details of the study selection are shown in the PRISMA flowchart in Figure 1. Specifically, the first author extracted key information from the full-text papers, which was then double-checked and validated by the second author before inputting into an Excel table. The extracted data included citation of the paper, country where the study was conducted, study design, setting, total number of participants, mean age, disease, intervention provider, intervention delivered to the intervention and control groups, frequency and duration of the intervention and follow-up outcomes.

### Quality assessment of included studies

Given that all studies identified were RCTs, the recommended tool by Cochrane, specifically version 2 of the Cochrane risk-of-bias tool for randomised trials (RoB 2), was used to assess the RoB in the trials included in this study (Sterne et al., 2019). The tool evaluates five domains: bias arising from the randomisation process, deviations from intended interventions, missing outcome data, outcome measurement and selection of the reported result. Each domain was scored as low RoB, unclear risk or high RoB. Studies were excluded if two or more domains were assessed as high RoB. During the methodological assessment process, two authors were involved, and any discrepancies that arose were resolved through mutual discussion. No additional studies were excluded during the discussion process. At each stage of study selection, records were screened according to the predefined eligibility criteria, and a total of 13 studies were included in the final analysis.

### Statistical analyses

The standardised mean difference (SMD) for continuous outcomes was calculated to assess the effects of MBIs on pain, anxiety, depression, and quality of life in patients receiving palliative care, as different scales were used to measure each outcome (Borenstein et al., 2021; Chandler et al., 2019). Pooled SMDs were generated using a random-effects model (Chandler et al., 2019). Heterogeneity of each forest plot was estimated with the *I*^2^ statistic, with values ⩾50% indicating substantial heterogeneity (Higgins et al., 2003). Forest plots were generated for the meta-analyses. Egger’s regression test was performed to investigate the influence of publication bias on each pooled analysis (Egger et al., 1997). Additionally, leave-one-out sensitivity analysis, excluding the study with the highest weight in each forest plot, was conducted to assess the stability of the pooled SMD for each outcome (Iyengar and Greenhouse, 2009). All statistical analyses were performed using STATA 17, StataCorp LLC, United States.

### Study characteristics

Thirteen RCTs, involving 1056 patients in palliative care, conducted in Canada, Germany, Malaysia, the United Kingdom, and the United States were selected for this study. The patients receiving palliative care were primarily suffering from cancer and ranged in age from 44.5 (±7.7) to 69.2 (±2.5) years. The care model was delivered in various settings, including cancer centre, medical centre, clinic, hospice centre, and hospital. The intervention group received a variety of mindfulness interventions, such as mindful breathing, mindfulness-based supportive therapy, mindfulness exercises, mindfulness-based cognitive therapy, and meditation, whereas all control groups received usual care apart from one study (Tan et al., 2024), which compared mindfulness-based supportive therapy with therapeutic listening focused on mindfulness. The interventions ranged in duration from 5 minutes to 2 hours per session, with follow-up periods varying from 4 weeks to 6 months. Detailed characteristics of the studies are provided in Table 1.

**Table 1.** Summary of included studies.

No	Author/year	Country	Setting	Participant’s			Intervention’s provider	Intervention types		Duration of intervention	Follow-up	Outcome/scale
				Total	Age (mean and SD, year), I/C	Type of disease		Experimental group	Control group			
1	Bower et al. (2021)	United Sates	Cancer Centre	247	44.5 (7.7)/45.9 (5.6)	Breast cancer	Trained nurses or physicians	Received theoretical materials on mindfulness, relaxation and the mind–body connection; experiential practice of meditation; and psychoeducational	NA	2-hour sessions	6 months	Depressive symptoms (CES-D), fatigue (FSI), insomnia (ISI)
2	Galfin et al. (2012)	United Kingdom	Hospices centre	34	67.32 (11.60)/62.67 (11. 86)	NA	NA	Received a training and digital recording of the exercise to practice each day and a booklet explaining the training	Receiving usual hospice care	30 minutes	4 weeks	Anxiety (GAD-7), depression (BDI), quality of life (WHOQOL-BREF)
3	Guan et al. (2021)	Malaysia	Hospital	60	47.03 (16.46)	Cancer	NA	Received 5 minutes of mindful breathing	Received 5 minutes of normal listening	5–10 minutes	NA	Pain (VAS)
4	Kubo et al. (2024)	United Sates	Medical Centre	142	65.8 (8.8)/67.1 (10.4)	Cancer	NA	Received mindfulness-based intervention	received usual care	10–20 minutes	NA	Depression, anxiety, quality of life
5	Lim et al. (2021)	Malaysia	Medical Centre	60	55.4 (14.0)/56.1 (15.6)	NA	NA	Received of 5 minutes of mindfulness	Received 5 minutes of supportive listening	5 minutes	NA	Suffering (Suffering Pictogram), quality of life (FACIT-Sp12 Version 4)
6	Look et al. (2021)	Malaysia	Medical Centre	40	66.7 (3.22)/69.2 (2.5)	Cancer	NA	Received single session of 20 minutes mindful breathing	Received standard care	20 minutes	NA	Pain, tiredness, drowsiness, nausea, depression, anxiety, well-being (ESAS)
7	Ng et al. (2016)	Malaysia	Medical Centre	60	47.03 (16.46)	Cancer	NA	Received 5 minutes of mindful breathing	Received 5 minutes of normal listening	20 minutes	NA	Distress (distress thermometer)
8	Speca et al. (2000)	Canada	Clinic	89	NA	Cancer	NA	Received mindfulness meditation	Received usual care	90 minutes	7 weeks	Depression (POMS), anxiety (POMS)
9	Tan et al. (2023)	Malaysia	Medical Centre	73	60.7 (12.7)/56.5 (17.6)	Cancer	Medical Doctor	Received mindfulness-based supportive therapy	Received 30 minutes of supportive listening	30 minutes	NA	Depression (HADS), quality of life (FACIT-Sp12 Version 4)
10	Teo et al. (2020)	Singapore	Oncology clinics	85	60.0 (12.9)/55 (9.5)	Breast Cancer	NA	Received cognitive-behavioural strategies with mindfulness and values-based activity	NA	1 hour	8 weeks	Anxiety (HADS), depression (HADS), pain severity and pain disability, fatigue (seven-item PROMIS Fatigue-Short Form)
11	Warth et al. (2015)	German	Hospital	84	63.0 (13.4)	Cancer	Music therapists	Received short mindfulness exercise, accompanied by soft monochord sounds	Received the same duration but with no musical content or therapeutic relationship	5–15 minutes	NA	pain (VAS), fatigue (NA), quality of life (NA)
12	Warth et al. (2020)	German	Hospital	42	65.9 (13.02)	Cancer	NA	Received a short breathing exercise and guided body scan meditation for supine positions	NA	20 minutes	NA	Quality of life (VAS_WELL), distress (VAS_STRESS)
13	Yik et al. (2020)	Malaysia	Medical Centre	40	56.7 (12.9)/58.3 (13.7)	Cancer	NA	Received brief mindfulness practice	Received 5 minutes of active listening	5–10 minutes	NA	Quality of life (FACIT-Sp12 Version 4)

NA: not available; BDI: Beck Depression Inventory; BPI: Brief Pain Inventory; CES-D: Centre for Epidemiologic Studies-Depression Scale; ESAS: Edmonton Symptom Assessment Scale; FACIT-Sp12 Version 4: Functional Assessment of Chronic Illness Therapy; FSI: fatigue symptom inventory; GAD-7: Generalised Anxiety Disorder-7; HADS: Hospital Anxiety and Depression Scale; ISI: insomnia severity index; POMS: Psychiatric Outpatient Mood Scale; VAS: Visual Analogue Scale; WHOQOL-BREF: World Health Organization Quality of Life-Short Version.

## Results

### Effects of MBIs in patients receiving palliative care

#### Pain

**Figure 2.**
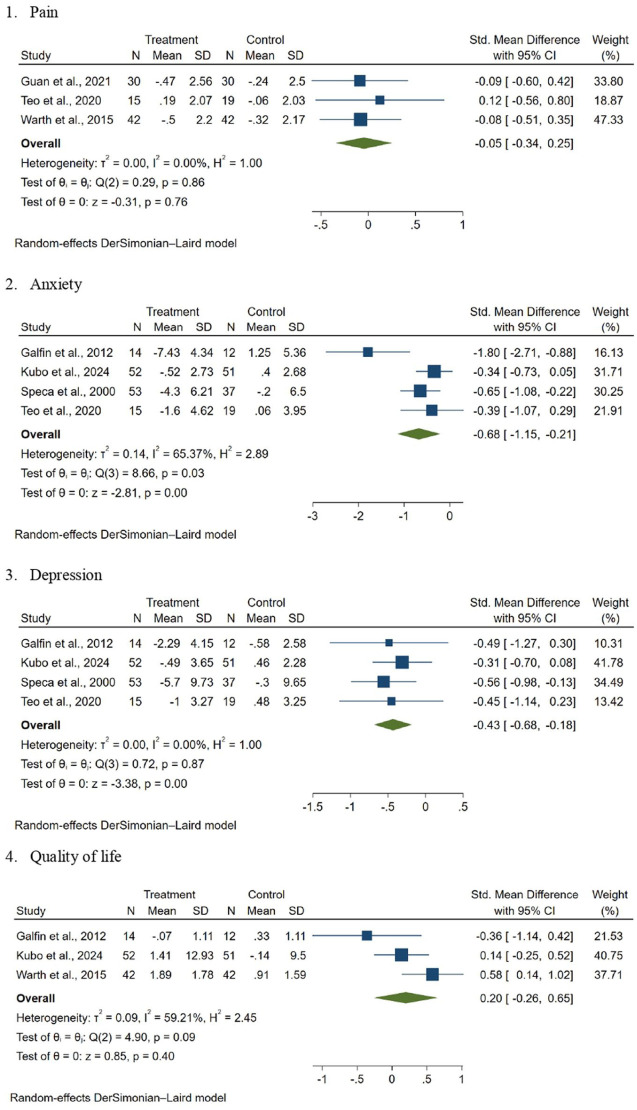
Forest plot of effect of mindfulness-based interventions in patients receiving palliative care. (1) Pain, (2). Anxiety. (3). Depression. (4). Quality of life.

Three RCTs reported the effects of MBIs on pain using the Visual Analogue Scale and the Brief Pain Inventory (Guan et al., 2021; Kubo et al., 2024; Warth et al., 2015). The pooled SMD was −0.05 (95% confidence interval [CI] = −0.34 to 0.25, *p* = 0.40, *I*^2^ = 0.00%; Figure 2(1)), indicating that MBIs did not significantly reduce pain in patients receiving palliative care.

#### Anxiety

Four RCTs reported the effects of MBIs on anxiety using the Generalised Anxiety Disorder-7, the Hospital Anxiety and Depression Scale and the Psychiatric Outpatient Mood Scale (Galfin et al., 2012; Kubo et al., 2024; Speca et al., 2000; Teo et al., 2020). The pooled SMD was −0.68 (95% CI = −1.15 to −0.21, *p* < 0.001, *I*^2^ = 65.37%%; Figure 2(2)), indicating that MBIs significantly reduced anxiety in patients receiving palliative care.

#### Depression

Four RCTs reported the effects of MBIs on depression using the Beck Depression Inventory, the Hospital Anxiety and Depression Scale and the Psychiatric Outpatient Mood Scale (Galfin et al., 2012; Kubo et al., 2024; Speca et al., 2000; Teo et al., 2020). The pooled SMD was −0.43 (95% CI = −0.68 to −0.18, *p* < 0.001, *I*^2^ = 0.00%%) (Figure 2(3)), indicating that patients receiving palliative care who underwent MBIs had fewer symptoms of depression compared to the control group.

#### Quality of life

Three RCTs reported the effects of MBIs on quality of life using the World Health Organization Quality of Life-Short Version and the Functional Assessment of Chronic Illness Therapy (Galfin et al., 2012; Kubo et al., 2024; Warth et al., 2015). The pooled SMD was 0.20 (95% CI −0.26 to 0.65, *p* = 0.40, *I*² = 59.21%) (Figure 2(4)), indicating that MBIs did not significantly improve quality of life of patients receiving palliative care.

### Risk of bias

The overall RoB in all included trials was considered to be low (see Supplemental Appendix C). Potential publication bias was identified regarding deviations from intended interventions due to issues with blinding, which could have resulted in variability of outcome effects across studies and biased the true effect of the intervention. However, Egger’s regression test indicated that the influence of publication bias was minor in each pooled analysis: pain (*p* = 0.71), anxiety (*p* = 0.21), depression (*p* = 0.82) and quality of life (*p* = 0.447).

### Sensitivity analysis

Leave-one-out sensitivity analysis for each pooled outcome, after excluding the most influential trial from the pooled result, revealed that the outlier study did not significantly influence the overall stability of each pooled SMD estimate in the meta-analysis: pain (*p* = 0.90), anxiety (*p* = 0.66), depression (*p* = 0.68) and quality of life (*p* = 0.95)

## Discussion

### Impact on pain

The effect of MBIs on pain among palliative care patients, including cancer patients with advanced disease, was evaluated in three RCTs (Guan et al., 2021; Kubo et al., 2024; Warth et al., 2015). The results indicate that MBIs do not significantly reduce pain in these patients. This aligns with a meta-analysis synthesis conducted by Sharpe et al. (2024), which concluded that while MBIs can lead to small improvements in pain intensity, these changes are not substantial enough to support the recommendation of MBIs as a comprehensive solution for pain management. Additionally, Zhang et al. (2019) conducted a systematic review and meta-analysis of MBSR and found limited effectiveness of MBIs in reducing pain, specifically among cancer patients, with the changes being statistically non-significant. Stadnyk et al. (2023) also emphasised the challenges associated with measuring pain, highlighting that it is a subjective and individualised experience, which complicates the assessment of intervention outcomes. Furthermore, the lack of significant pain reduction can be attributed to the fact that most patients in palliative care programmes are already receiving effective pain management through active control, as revealed in one included study by Warth et al. (2015), where the baseline pain severity was not significant. Overall, these studies indicate that while MBIs may provide some comfort and support, they should be regarded as supplementary rather than primary treatments for pain and should be integrated with other pain management strategies to enhance patient outcomes.

### Impact on anxiety and depression

The results of reviews on MBIs for reducing anxiety and depression symptoms in palliative patients, including cancer patients, are consistent with existing systematic reviews and meta-analyses. These findings demonstrate significant reductions in anxiety and depression symptoms across various studies, with medium to large effect sizes (Chayadi et al., 2022; Fan et al., 2023; Hofmann and Gómez, 2017; Wang et al., 2024; Yu et al., 2023). However, among the four studies that measured both depression and anxiety, only one study (Galfin et al., 2012) found non-significant results for depression. There were two notable differences in this study compared to the other three. Firstly, it focused exclusively on patients with clinically significant psychological distress, unlike the other three studies. Secondly, the MBI method used in that study was a brief guided self-help approach, which may not be as effective as the MBIs that were not self-administered, such as those using specific MBSR (Speca et al., 2000), a combination with cognitive-behavioural therapy (Teo et al., 2020) and online MBI modifications (Kubo et al., 2024). Previous review papers have validated these specific MBIs, showing that MBSR is superior to standard treatment in reducing symptoms of anxiety and depression (Wu et al., 2022; Xunlin et al., 2020; Yu et al., 2023), and online MBIs have been effective in reducing depression in cancer patients (Fan et al., 2023). This evidence indicates that MBIs can be a valuable adjunctive therapy for patients receiving palliative care, providing relief from anxiety and depression.

It is important to note that the observed effects likely reflect reductions in specific symptoms of anxiety and depression rather than alleviation of the underlying psychological conditions themselves. MBIs may therefore support symptom management rather than function as a definitive treatment for clinical anxiety or depressive disorders.

### Impact on quality of life

Regarding quality of life, the effect of MBIs was not significant. Two of the three studies examined concluded that mindfulness did not improve quality of life (Galfin et al., 2012; Warth et al., 2015). However, one study (Kubo et al., 2024) did show some improvement; however, this study had limitations and the results cannot be generalised, as it was only a pilot study providing preliminary evidence on the feasibility of conducting mindfulness interventions. Therefore, further rigorous research is needed. This indicates that while mindfulness may improve certain psychological outcomes such as anxiety and depression, it does not necessarily lead to meaningful enhancement in the overall quality of life for patients in palliative care.

### Broader interpretation and clinical meaning

This systematic review and meta-analysis investigated the effects of MBIs on pain, anxiety, depression, and quality of life in patients receiving palliative care. The findings suggest that MBIs significantly reduced symptoms of anxiety and depression in these patients. However, no significant effects were observed in terms of pain reduction and improvement in quality of life. These results provide important insights into the specific areas where MBIs can be beneficial, while also highlighting the need for a more nuanced understanding of their limitations. In addition to symptom-focused outcomes, mindfulness-based interventions may hold broader value in palliative care by supporting patients’ sense of agency and self-management. Several theoretical and empirical works suggest that MBIs promote feelings of autonomy, mastery and personal control, which are particularly meaningful for individuals facing advanced illness (Garland et al., 2015; Kabat-Zinn, 2013). In palliative contexts, where loss of control is a common source of psychological distress, self-directed practices such as mindful breathing, moment-by-moment awareness, and non-reactive acceptance may help patients regain a sense of participation in their own care trajectory (Rodriguez, 2022; Ruskin et al., 2021). The importance of interventions that patients can practice independently without reliance on pharmacological management or clinician-mediated strategies has been emphasised as a vital component of dignity-conserving and person-centred palliative models (Chochinov, 2022). Therefore, although the quantitative effects of MBIs on pain and quality of life remain uncertain, their potential role as self-managed tools that enhance agency and emotional coping may represent an additional, clinically meaningful benefit that merits further attention in future trials.

MBIs appear to be a valuable adjunctive therapy for alleviating anxiety and depression in patients receiving palliative care, but further research is needed to explore their potential benefits for pain management and quality of life enhancement. Beyond the measurable clinical outcomes evaluated in this review, it is important to consider that MBIs may alleviate distress not only through symptom reduction but also by enhancing patients’ sense of agency in managing the psychosocial impacts of advanced illness. Mindfulness practices encourage self-directed engagement, moment-to-moment awareness and acceptance-based coping, which may empower patients to feel a greater sense of control and participation in their care despite the constraints of terminal disease (Garland et al., 2015; Kabat-Zinn et al., 1992). Although agency and self-management were not assessed as explicit outcomes within the included trials, these constructs are integral to the therapeutic mechanisms of MBIs and may contribute indirectly to improvements in emotional well-being and perceived quality of life. Evidence from qualitative and mixed-methods research suggests that palliative care patients often experience mindfulness as a means of regaining personal autonomy, reducing helplessness, and reframing their relationship with suffering (Rodriguez, 2022; Ruskin et al., 2021). Thus, while our meta-analysis did not identify statistically significant improvements in quality of life, it remains possible that MBIs support important experiential outcomes such as enhanced agency and self-efficacy that may not be fully captured by conventional QoL scales but are nevertheless meaningful within palliative care contexts. Future trials would benefit from incorporating validated measures of agency, self-management and existential coping to more comprehensively evaluate the broader benefits of MBIs.

### Implications for nursing practice, education, policy and research

The findings of this study carry implications for nursing practice, nursing education, healthcare policy, and future research in palliative care. For nursing practice, MBIs may serve as accessible, low-risk, and patient-centred approaches that nurses can incorporate into supportive care to help manage psychological distress and promote emotional well-being. For nursing education, integrating foundational concepts of mindfulness and evidence-based nonpharmacological interventions into training programmes may better prepare nurses to guide patients in these techniques and to recognise when such interventions may be beneficial. From a policy standpoint, MBIs could be considered for inclusion in supportive care pathways as low-cost, self-managed strategies that complement existing treatment options and enhance holistic care delivery. For future research, the limited number of trials, heterogeneity of interventions, and absence of paediatric studies highlight the need for standardised MBI protocols and more rigorous evaluation of both symptom outcomes and experiential constructs such as agency coping, and self-management. In education, incorporating mindfulness concepts into palliative care training may help clinicians introduce MBIs appropriately and support patients in using them as adjunctive tools to address psychosocial distress. Moreover, aligning MBI training with foundational nursing theories such as Watson’s Human Caring Theory and related humanistic frameworks may further strengthen nursing curricula by emphasising mindful presence, authentic relational engagement, and holistic support. Because MBIs cultivate awareness, compassion and nonjudgmental presence, they operationalise core caring healing processes central to transpersonal nursing practice. Integrating this theoretical grounding into education may better equip nurses to deliver MBIs in ways that uphold the values of dignity, personhood, and relational care that these theories advocate.

### Strengths and limitations

This systematic review and meta-analysis has several key strengths. It provides the most up-to-date synthesis of RCT evidence on MBIs in palliative care, drawing on 13 trials identified through a comprehensive search of six databases and grey literature, without language or geographic restrictions. The review followed a preregistered protocol, adhered to PRISMA guidelines and employed rigorous methods, including risk-of-bias assessment, random-effects meta-analysis, publication bias testing, and sensitivity analyses, enhancing the reliability and transparency of the findings. By focusing specifically on patients receiving palliative care and reporting outcome-specific estimates for pain, anxiety, depression, and quality of life, the review offers clinically relevant insights for nursing practice, education, and policy.

Despite these strengths, several limitations should be noted. Although MBIs demonstrated statistically significant reductions in anxiety and depression, these findings were derived from a small number of heterogeneous trials that varied in mindfulness modality, intervention duration, delivery format, and outcome measures. Inconsistent results for pain and quality of life indicate that MBIs cannot yet be considered broadly effective across symptom domains. Consequently, the findings should be interpreted as preliminary and context dependent. Across all outcomes, only three to four trials contributed data, limiting statistical power and generalisability. Substantial heterogeneity likely influenced effect sizes and contributed to mixed results. Additionally, all included studies enrolled adults, with no trials involving children or adolescents, limiting applicability to paediatric populations. More rigorous, methodologically standardised, and age-inclusive trials are needed to strengthen the evidence base and reduce heterogeneity in future research.

**Key points for policy, practice and/or research**

- Mindfulness-based interventions (MBIs) significantly reduce anxiety and depression in palliative care patients.
- MBIs provide a feasible, non-pharmacological adjunct to holistic palliative care nursing models.
- Further rigorous trials are required to assess effects on pain and overall quality of life.

## Conclusion

In conclusion, this systematic review and meta-analysis highlight that MBIs offer significant benefits in reducing anxiety and depression among patients receiving palliative care, although their effects on pain management and quality of life remain inconclusive. Given the small number of available trials for each outcome, the variability in intervention types and the heterogeneity across studies, our findings should be interpreted with caution. Importantly, the current body of evidence does not allow for strong or definitive conclusions regarding the overall effectiveness of MBIs in palliative care. Despite showing promise for alleviating psychological distress, MBIs do not appear to significantly improve pain or overall quality of life, emphasising the need for a complementary approach in these areas. Accordingly, our revised conclusion acknowledges that the evidence base is limited and heterogeneous, and MBIs should be considered only as a potentially supportive intervention until more rigorous and adequately powered trials are conducted. Future studies should standardise MBI protocols, increase sample sizes, and implement rigorous blinding to provide more definitive evidence and enhance the reliability of findings.

## Supplemental Material

sj-docx-1-jrn-10.1177_17449871261419719 – Supplemental material for Mindfulness-based interventions for pain, anxiety, depression, and quality of life in patients receiving palliative care: a meta-analysis Supplemental material, sj-docx-1-jrn-10.1177_17449871261419719 for Mindfulness-based interventions for pain, anxiety, depression, and quality of life in patients receiving palliative care: a meta-analysis by Ice Septriani Saragih, Dame Elysabeth Tuty Arna Uly Tarihoran, Huey-Ming Tzeng and Ita Daryanti Saragih in Journal of Research in Nursing
